# Confocal microscopy for astrocyte in vivo imaging: Recycle and reuse in microscopy

**DOI:** 10.3389/fncel.2013.00051

**Published:** 2013-04-29

**Authors:** Alberto Pérez-Alvarez, Alfonso Araque, Eduardo D. Martín

**Affiliations:** ^1^Instituto Cajal, Consejo Superior de Investigaciones CientíficasMadrid, Spain; ^2^Parque Científico y Tecnológico de Albacete, Instituto de Investigación en Discapacidades Neurológicas, Universidad Castilla-La ManchaAlbacete, Spain

**Keywords:** *in vivo*, imaging, astrocyte, two-photon, confocal microscopy, cranial window, intravital, glia

## Abstract

*In vivo* imaging is one of the ultimate and fundamental approaches for the study of the brain. Two-photon laser scanning microscopy (2PLSM) constitutes the state-of-the-art technique in current neuroscience to address questions regarding brain cell structure, development and function, blood flow regulation and metabolism. This technique evolved from laser scanning confocal microscopy (LSCM), which impacted the field with a major improvement in image resolution of live tissues in the 1980s compared to widefield microscopy. While nowadays some of the unparalleled features of 2PLSM make it the tool of choice for brain studies *in vivo*, such as the possibility to image deep within a tissue, LSCM can still be useful in this matter. Here we discuss the validity and limitations of LSCM and provide a guide to perform high-resolution *in vivo* imaging of the brain of live rodents with minimal mechanical disruption employing LSCM. We describe the surgical procedure and experimental setup that allowed us to record intracellular calcium variations in astrocytes evoked by sensory stimulation, and to monitor intact neuronal dendritic spines and astrocytic processes as well as blood vessel dynamics. Therefore, in spite of certain limitations that need to be carefully considered, LSCM constitutes a useful, convenient, and affordable tool for brain studies *in vivo*.

## INTRODUCTION

The combination of fluorescence techniques with two-photon laser scanning microscopy (2PLSM) has become the tool of choice for *in vivo* brain imaging because high-resolution images can be obtained at relatively high depth (>500 μm) from the tissue surface (Theer et al., 2003; Helmchen and Denk, 2005). It employs ultrashort infrared laser pulses for fluorophore excitation that yield low light scattering by the tissue sample or living brain. In addition, the non-linear nature of 2PLSM excitation grants that detected photons come from fluorophore emission exclusively at the focal plane (Svoboda and Yasuda, 2006). In contrast, laser scanning confocal microscopy (LSCM) employs single-photon excitation, which is more sensitive to scattering (Centonze and White, 1998). It relies on a pinhole to reject fluorescence from out-of-focus locations to create high-resolution contrast images of relatively superficial areas (<100 μm from the surface). Interestingly, due to the use of shorter light wavelengths the resolution obtained with LSCM is better as the point spread function (e.g., pattern of diffracted light from a subresolution point-source which gives a measure of the smallest objects that can be resolved) is smaller (~300 nm in *xy* axis; ~900 nm in *z* axis; Abbe, 1873, 1874; Cole et al., 2011).

Although the commercial availability of two-photon laser scanning microscopes has led to their widespread use, their overall cost may still be prohibitive for some laboratories to perform brain studies *in vivo*. However, single-photon LSCM is more widely available and has also been technologically improved (e.g., being employed in neurosurgery for intraoperative diagnosis and applied for *in vivo* research in moving animals; Jung et al., 2004; Kedrin et al., 2008; Eschbacher et al., 2012; Ritsma et al., 2012).

Astrocytes and their thin processes maintain close structural and functional interactions with neurons and synapses (Ventura and Harris, 1999; Bushong et al., 2002). They respond to synaptic activity (Perea and Araque, 2005) and influence synaptic transmission (Fiacco and McCarthy, 2004; Perea and Araque, 2007; Di Castro et al., 2011; Panatier et al., 2011) and plasticity (Henneberger et al., 2010; Takata et al., 2011; Navarrete et al., 2012). Also, they enwrap blood vessels with specialized processes termed endfeet, which play relevant roles in controlling local metabolic and energetic demands through the so-called neuro-glio-vascular coupling (Zonta et al., 2003; Mulligan and MacVicar, 2004; Metea and Newman, 2006; Takano et al., 2006; Gordon et al., 2008; Attwell et al., 2010). Monitoring *in vivo* these structural and functional relationships between astrocytes and neurons may provide relevant information about their actual conditions and properties in intact or minimally perturbed preparations. While 2PLSM has been successfully applied to address these issues (Hirase et al., 2004; Takano et al., 2006; Wang et al., 2006; Schummers et al., 2008; Takata et al., 2011), LSCM may also be useful for these purposes (Mishra et al., 2011; Navarrete et al., 2012; Srienc et al., 2012).

In this article we show that the combination of an optimized surgical procedure with intravital staining of astrocytes and LSCM represents a suitable approach for imaging *in vivo* the subcellular structure of astrocytes and neurons, monitoring calcium transients in the astrocytic soma and processes, and visualizing blood vessel dynamics. We additionally provide a detailed description of the methodology used to carry out *in vivo* imaging in the mouse brain cortex using LSCM.

## MATERIALS AND METHODS

### MATERIALS

#### Reagents

• HEPES-buffered saline (in mM: NaCl 140, KCl 5, MgCl_2_ 1, CaCl_2_ 2, EDTA 1, HEPES potassium 8.6, glucose 10)
• 0.9% (w/v) NaCl (saline)
• Urethane (Sigma, Madrid, Spain). Dissolve in saline.
• Fluo-4 AM (Life Technologies, Barcelona, Spain). Dissolve 50 μg in 4 μl pluronic [(Life Technologies, 20% in dimethyl sulfoxide (DMSO)]. Add 46 μl of HEPES-buffered saline to obtain a 1 μg/μl final concentration. Vortex to achieve dissolution.
• Sulforhodamine 101 (SR101; Sigma, Madrid, Spain). Dissolve in saline according to the weight of the animal (100 mg/kg).
• Fortex dental cement (Facident, Barcelona, Spain).
• Low melting point agarose (1% in saline; Sigma, Madrid, Spain).

#### Equipment

• Stereotaxic device (ASI Instruments, Warren, MI, USA)
• Mouse Adaptor (Stoelting Co, IL, USA)
• Aluminum cranial frame
• Electronic control for heat pad (Cibertec, Madrid, Spain)
• Heat pad (RS Amidata, Madrid, Spain)
• Rectal probe (Technomed Europe, Maastricht, The Netherlands, Cat No. TP/YSI402)
• Drill Volvere Vmax NE120 (Nakanishi Inc., Kanuma, Japan)
• Burrs (FST, Heidelberg, Germany, Cat No. 19007-14/07)
• Stainless Steel Mounting Screws 00-96 X 1/16 (Plastic One, VA, USA)
• Drill holder (Plastic One, VA, USA, Cat No. DH 1)
• Drill bit (Plastic One, VA, USA, Cat No. D #60)
• Screwdriver (Plastic One, VA, USA, Cat No. SD 96)
• Surgical blade
• Set of surgical forceps (FST, Heidelberg, Germany)
• Scissors [Vannas and common type; FST (Fine Science Tools), Heidelberg, Germany]
• Spatula
• Cotton
• Glass coverslips (5–6 mm diameter, 0.15 mm thickness; Menzel, Braunschweig, Germany)
• Syringe (10 ml)

#### Microscope

• Olympus FV300 laser scanning confocal system coupled to an Olympus BX61WI upright microscope (Olympus, Tokyo, Japan)
• Lasers: Ar 488 and HeNe 543 (2.5 and 0.5 mW, respectively at the objective back focal plane; CVI Melles Griot, Cambridge, UK)
• Fluoview software for acquisition (Olympus, Tokyo, Japan)
• Water immersion Olympus LUMPLFL 60XW/IR objective (0.9NA; Olympus, Tokyo, Japan)
• Scientifica Movable Top Plate (Scientifica, Uckfield, UK)
• PMI-100 pressure injector (Dagan, MN, USA)
• Axon Digidata 1322A (Molecular Devices, CA, USA)
• pClamp software (Molecular Devices, CA, USA)

### MICE

We employed Thy-1 GFP-M transgenic mice (The Jackson Laboratory, ME, USA), which express green fluorescent protein (GFP) under the Thy1 promoter (Feng et al., 2000), to visualize dendrites projecting from layer V pyramidal neurons. All the procedures for handling and sacrificing animals followed the European Commission guidelines (86/609/CEE).

### EQUIPMENT SETUP

#### Cranial frame

**FIGURE 1:**
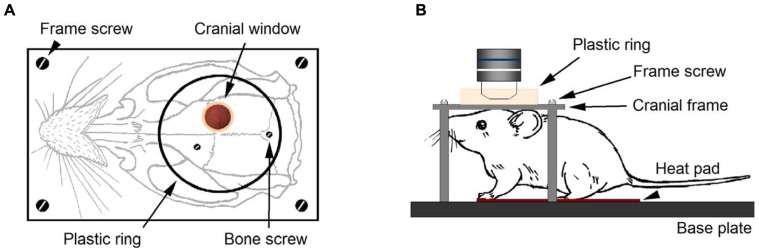
**Experimental setup for *in vivo* imaging with LSCM**. After performing a cranial window on a Thy-1 GFP-M mouse, the cranial frame **(A)** is secured onto the skull with dental cement. Two cranial screws, eventually embedded in cement, provide additional grip of the frame to the skull. A plastic ring delimits the area of the skull where cement is applied. Four screws secure the cranial frame to the heavy aluminum base plate **(B)**. During imaging, the animal head remained firmly secured to the base plate under the microscope objective. The mouse rested on the heat pad for temperature control and breathing was checked to be rhythmic and effortless.

The custom-designed frames consisted of a heavy aluminum base plate (7 cm × 13 cm × 1 cm) and one light aluminum cranial frame (2 × 3.5 cm) for the cranial window. The latter has a central circular hole (10 mm diameter) and four holes in the corners to fit four M4 screws that will fix this plate to the heavy aluminum base plate (**Figure 1A**). The imaging chamber consisted of a circular plastic ring glued to the frame and centered in the cranial window. This frame provides stability for preparation and avoids the mechanical interference by respiration-induced movements caused by chest motion during breathing. The frame was attached onto the skull with two stainless steel screws and dental cement (see **Figure 1A**). The heavy aluminum base plate with the animal and the cranial frame fixed to it was attached to a Scientifica electrophysiology movable top plate and the height of the stage was adjusted in order to place it below the microscope scan head (**Figure 1B**). We removed the condenser and its holder to avoid mechanical interference with the stage when moving to search for regions of interest. Our assembly proved to be very convenient in its use during experiments due to its ample working area, stability, and smooth micromanipulator movement.

### PROCEDURE

#### Presurgical preparation

1. Weigh the animal (4–12 weeks old).
2. Inject SR101 (100 mg/kg) intraperitoneally. Let the animal rest in the cage with food and water for 1 h.
3. Observe intense coloration of ears and paws after 30 min.
4. Anesthetize with an intraperitoneal injection of urethane (1.8 mg/kg).
5. After 5 min, check an effective anesthetized state monitoring for awareness signs such as whisker twitching, palpebral reflex, and respiration rate when pinching the tail or ears.
6. Connect the heating pad to the thermostat and set to 37°C.
7. Shave the coronal area of the head and put the animal on the heating pad.
8. Gently insert the tip of the rectal probe (use lubricant) to continuously monitor the animal temperature (37°C) and tape it to the tail.
9. Mount the animal onto the stereotaxic apparatus. Hold the animal head 1" high and slide one of the ear bars slowly into the ear canal until a little resistance is encountered and secure the screw. Then proceed with the other bar until the head rests on both (ear bar coordinates = 4 mm).
10. Lower the tooth bar and insert it gently into the animal mouth (tooth bar coordinates = 0 mm), so the head is horizontal and the animal breathes easily.

#### Surgical procedure

11. Clean the surgical area with a cotton pad soaked in saline.
12. With a surgical blade, make a rostrocaudal incision from the midline between the eyes to the back of the head and retract the skin to both sides using forceps.
13. After exposing the skull, use a spatula to gently scrape out the periosteum and adjoining connective tissue. In some cases, the right *temporalis* muscle was separated from the bone.
14. Locate the area of interest. In our case, the somatosensory area situated -1 mm posterior to the bregma and 3 mm lateral from midline.
15. Insert two supporting screws. Mark the skull surface where the screws are going to be located (one in the midline of occipital bone and one in the contralateral frontal bone). Start performing a hole with the electrical drill and, before complete perforation, change to a manual drill and slowly continue until the dura is reached.
16. Bottom of the hole looks pinkish. Partially screw the supporting screws.
17. Make a circular groove (4 mm diameter) on the skull surface circling the area of interest with the appropriate drill bit (FST Cat No. 19007-14) at constant speed (1300 rpm). Slowly drill in a circular fashion, stopping from time to time to wet the area with a drop of saline.
18. Gently lift up and remove the circular bone fragment with the forceps and without touching the brain surface.
19. With a Dumont 5 forceps, grab the meninges at the most caudal part and lift them 1–2 mm and employ the Vannas scissors to perform a cut following the circle of the cranial window.
20. Fluo-4 AM bulk loading. Drop 5–10 μl of Fluo-4 AM (1 μg/μl) forming a meniscus on the cranial window. If this is not possible, ensure that the exposed surface is always covered by a thin liquid layer of the mixture. Wait for 30 min. Skip this step if calcium imaging is not intended.
21. Rinse with two drops of saline and cover the surface with soaked cotton. Skip this step if calcium imaging is not intended.
22. Maintain a glass coverslip 1 cm above the cranial window in preparation for the next two steps.
23. Remove the soaked cotton and use a plastic Pasteur pipette to put a drop of agarose (1% in HEPES-buffered solution) on the cranial window. Test for adequate temperature.
24. Lower the coverslip until it touches the agarose and the borders of the cranial window. Maintain pressure for 2–5 min. After, remove agarose excess from the sides of the window and dry the skull surface.
25. Apply cement to the borders of the coverslip (1–2 mm) and the skull surface with a thin spatula. This will fix the coverslip to the skull and also prevent saline leaks into the cranial window which would result in movement during imaging.
26. Let dry (~10 min) and check for solidness.
27. Make sure the skull is dry, specially the area where the plate is going to be cemented.
28. Put the cranial frame on the skull, placing the cranial window in the middle of the central plate aperture (see **Figure 1A**).
29. Extend cement over the skull surface and the borders of the plate aperture. Also embed the screws and the cement surrounding the coverslip.
30. Let dry (~20 min) and check for solidness.
31. Loosen the ear bars and screw the cranial frame to the heavy aluminum base.
32. Move the base plate with the animal and the thermal blanket to the imaging stage.
33. Fix the base plate on the imaging stage and put 2 ml of saline on the coverslip.
34. Lower the objective and start imaging.

After experimentation the animal was sacrificed by cervical dislocation and the base plate carefully removed. The frame was wiped with acetone.

### PROCEDURES-POINTS TO CONSIDER

#### Movement

Like in 2PLSM techniques, in the experimental approach with LSCM the control of movement is critical to obtain a high quality image of the brain during data acquisition. There are two well-established main sources of movement: a large amplitude respiration-induced movement caused by chest motion during breathing and regular small amplitude pulsatile movement synchronized to the heart beat. To avoid respiration-induced movements, we have used a custom-designed frame that provides stability for experimental preparation. In addition, the vascular pulsatile movement was abolished by filling the craniotomy with agarose and attaching a coverslip to the skull with dental cement. If the sealing of the cranial window with dental cement leaks, or is deteriorated by any cause, it will notably reduce the stability of the preparation.

#### Surgical care

The optical clarity of the cranial window depends on the quality of the surgery and the technique is highly operator-dependent. Therefore, the outcomes are sometimes unpredictable. Preventing cerebral edema and reducing inflammation during the surgery is critical for successful experimentation. It may be appropriate to use dexamethasone by an intramuscular injection to the quadriceps reducing the cortical stress response during the surgery and prevent cerebral edema. During trepanation, excessive pressure should not be applied when drilling because this might puncture the skull and damage the dura. Check the thickness of the skull during craniotomy by pushing very gently on the cranial bone with a fine forceps. If the peripheral bone moves when lightly touched, it is ready to be removed. The next critical step arrives at the time of insertion of the forceps tip into the trabecular bone. Keep the tip in a horizontal position and try to avoid direct perforation of the thinned bone with the forceps, which could damage the dura. Best results are obtained when the skull bone is gently tugged laterally until the thinned bone tears at the bottom of the groove. In our experiments, we obtained better results removing the dura. With a sharp forceps, grab the dura at the most caudal part, lift them and employ the Vannas scissors to cut following the circle of the cranial window. As the dura is attached to the inner table of the cranium, some superficial capillaries might tear during removal of the cranial bone. Small focal bleeding typically disappears spontaneously or can be controlled by gently applying cotton soaked with saline over the exposed surface and waiting 2–5 min for hemostasis.

#### Stimulation paradigm

The left whiskers of the animal’s snout were stimulated with 100 ms puffs of air produced at 5 Hz by a pressure injector (Dagan, MN, USA) controlled by an Axon Digidata 1322A and pClamp software (Molecular Devices, CA, USA). Air was ejected at 1 bar pressure via capillary glass, attached to plastic tubing, positioned ~1 cm lateral and anterior to the animal’s nose to stimulate the whole left whisker pad. Pattern of stimulation was 5 Hz frequency (pulse width 100 ms) for 30 s. At the same time, tail pinching was performed at 2 Hz with steel forceps, providing a pairing protocol for astrocyte stimulation similar to that employed to induce cholinergic cortical plasticity (Takata et al., 2011).

## RESULTS

### *IN VIVO* IMAGING OF ASTROCYTES, NEURONS, AND BLOOD VESSELS USING LSCM

**FIGURE 2:**
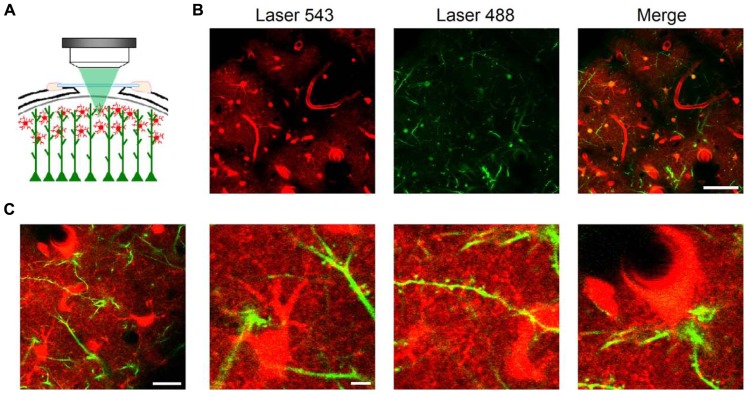
***In vivo* imaging of astrocytes, dendritic spines, and blood vessels using LSCM**. Schematic representation of an *in vivo* brain imaging experiment through a cranial window in a Thy-1 GFP-M mouse after intravital staining of astrocytes with SR101 **(A)**. At 60× magnification, astrocytic somata were clearly identified by intravital staining one hour after SR101 injection in single plane confocal images ~50 μm below the brain surface (**B**, left panel). GFP-expressing dendrites from layer V pyramidal neurons showed also distinct staining (**B**, central panel). Astrocytes were bulk loaded employing Fluo-4 AM and thus, also show fluorescence in the 488 channel (**B**, central and right panel). Large blood vessels project black shadows on the background since they are a major source of light scattering (Haiss et al., 2009). Astrocytes were observed projecting branches into the neuropil, contacting dendrites and their spines, and blood vessels through endfeet. Images taken at higher magnification in another area **(C)** show details of astrocytic somata and processes (**C**, second panel) contacting dendritic spines (**C**, third panel) and blood vessels (**C**, fourth panel). Scale bars: 50 μm in B; 20 μm (first panel) and 5 μm (second to fourth panels) in **(C)**.

To image astrocyte morphology *in vivo*, we performed a cranial window on anesthetized mice (**Figures 1** and **2A**) and took advantage of the fluorescent dye SR101 following a slightly modified intravital method recently reported (Appaix et al., 2012; see Methods). A single intraperitoneal injection of SR101 (100 mg/kg), usually before surgery, was administered to the animal. SR101 proved to be a good contrast agent which allowed us to discern clearly the brain blood vessels, from minutes to several hours after injection. Clear astrocyte staining was observed 40–60 min after injection. Astrocytes have been shown to selectively take up SR101 *in vivo* (Nimmerjahn et al., 2004; Appaix et al., 2012). Although the mechanism of uptake is still unknown, there is evidence showing that metabolites such as glucose and sulforhodamine spread efficiently across astroglial networks through gap junctions present in the astrocytic membrane (Rouach et al., 2008). SR101 taken from blood vessels by astrocytic endfeet were observed to spread with time throughout the astrocytic syncytium, reaching a maximum staining in about 1–2 h. Astrocytic somata were clearly identified as star-like cells, forming non-overlapping domains (Bushong et al., 2002) projecting several branches into the neuropil and contacting blood vessels and neuronal dendrites (**Figure 2**).

For simultaneous visualization of neurons, we used Thy-1 GFP-M mice, in which a subset of projecting neurons selectively expresses the GFP (Feng et al., 2000; **Figures 2A,B**). Both SR101 in astrocytes and GFP expressed in dendrites mainly from layer V pyramidal neurons provided a very strong signal-to-noise ratio which allowed us to maintain confocality (pinhole Airy units = 1) at low laser power below the intact brain surface (usually ~50 μm).

Therefore, following the methodology described in detail below, astrocytes, neurons, and blood vessels located near the brain surface can be monitored *in vivo* with high spatial resolution (~300 nm) using LSCM.

### *IN VIVO* IMAGING OF ASTROCYTIC PROCESSES AND DENDRITIC SPINES

Using the previous configuration at higher magnifications, we were able to image with subcellular resolution astrocytic processes and dendritic spines, i.e., two partners of the Tripartite Synapse (Araque et al., 1999; Perea et al., 2009). In spite of maintaining the laser power at relatively low levels (~0.5 mW for each laser line) to prevent photobleaching and photodamage, we were able to obtain high contrast images up to ~100 μm below the brain surface. In confocal microscopy, images beyond that depth are seriously limited by the increased and inherent light scattering (loss of ballistic photons and rejection of scattered ones by the pinhole). We typically obtained our images ~50 μm below the brain surface, which coincides with depths reported by many laboratories employing 2PLSM for *in vivo* recordings of astrocyte and neuronal dendrite morphology along with blood vessel integrity and dynamics (Schaffer et al., 2006; Takano et al., 2006, 2007; Mostany et al., 2010; Sigler and Murphy, 2010). Therefore, while 2PLSM allows deeper imaging, LSCM can be suitably used to monitor thin astrocytic processes and dendritic spines to extract relevant pathophysiological data (**Figures 2B,C**).

### *IN VIVO* IMAGING OF ASTROCYTE CALCIUM DYNAMICS

**FIGURE 3:**
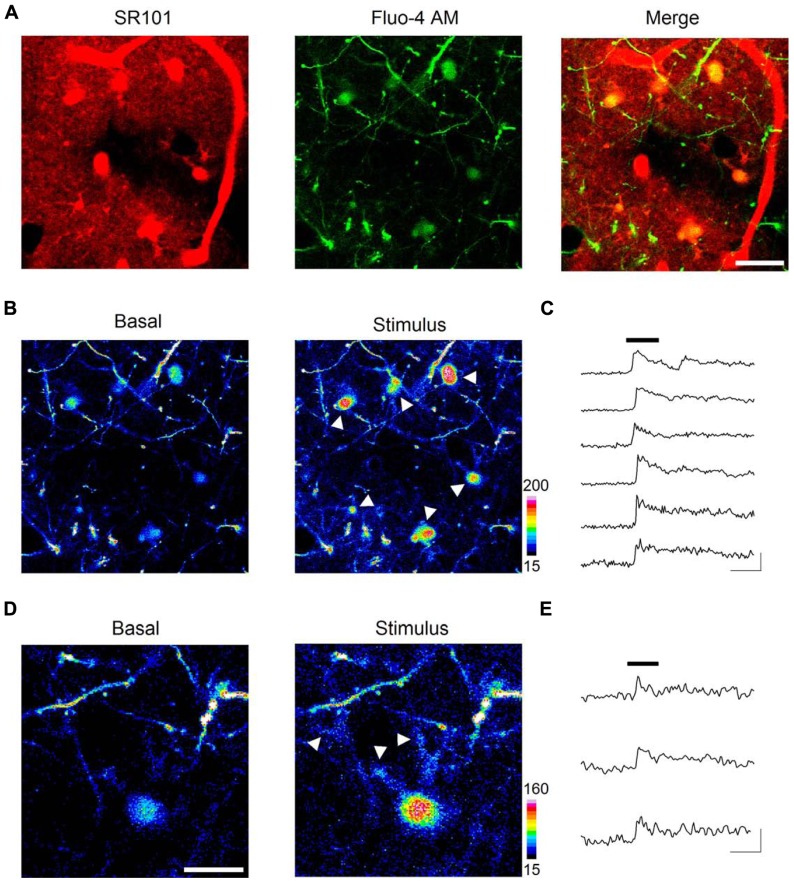
***In vivo* imaging of astrocyte calcium dynamics**. Intravitally stained astrocytes (**A**, left panel) were bulk loaded with the calcium indicator Fluo-4 AM (**A**, central panel), showed as areas of colocalization between SR101 and Fluo-4 AM in the merged image of a single confocal plane (**A**, right panel). Astrocyte basal calcium levels (**B**, left panel) increased upon sensory stimulation (see arrows in **B**, right panel). Time lapse recordings of cytosolic calcium in astrocytes reveal a strong elevation shortly after (~4 s) the onset of the 30 s sensory stimulus (black bar) and a slow recovery after cessation **(C)**. Sensory stimulation evoked calcium increases not only at astrocytic somata but also at discrete regions such as distal astrocytic processes (see arrows in **D**, right panel). Panel **(E)** shows time lapse recordings from those regions. Scale bars: 20 μm in **(A)** and **(B)**; 10 μm in **(D)**; 30 s, 100% ΔF in **(C)** and **(E)**.

Beyond obtaining structural images, we also aimed to monitor astrocyte intracellular calcium levels, which represent the basis of the astrocyte calcium excitability (Perea and Araque, 2005). Hence, we employed intravital astrocyte staining with SR101 (to identify astrocytes) along with Fluo-4-AM bulk loading (see Methods) of the barrel cortex in Thy-1 GFP-M mice (to identify dendritic spines; **Figure 3A**). Sensory stimuli of whiskers and tail (see Methods) induced intracellular calcium transients at the astrocytic somata (**Figures 3B,C**). These calcium elevations were recorded ~50 μm below the intact brain surface, which would correspond to layer I of the primary somatosensory cortex. These results are in close agreement with previous *in vivo* studies using 2PLSM in this cortical layer that documented calcium elevations in the soma of astrocytes (Takano et al., 2006, 2007; Takata and Hirase, 2008; Takata et al., 2011) as well as changes in blood vessel diameter upon electrical and sensory stimulation (Takano et al., 2006, 2007). Sensory stimulation-evoked calcium elevations have also been recorded in the hippocampal astrocytes after decortication with LSCM (Navarrete et al., 2012).

Interestingly, we further observed sensory stimulation-evoked astrocyte calcium elevations not only in the soma but also in processes located in close apposition to identified dendritic spines (i.e., GFP-expressing dendrites projecting from layer V neurons), where most excitatory terminals establish synaptic contacts (**Figures 3D,E**).

Taken together, these results support the suitability of LSCM to study physiological calcium signaling in astrocytes *in vivo*, and reveal the existence of localized subcellular microdomains in astrocytes that respond to sensory stimulation in the live animal.

### *IN VIVO* IMAGING OF BLOOD VESSEL DYNAMICS

Blood flow regulation in the brain is crucial for the adequate metabolic and oxygen supply to neurons in specific brain regions, and astrocytes are recognized to be involved in the control of functional hyperemia, i.e., changes in microvessel diameter and associated blood flow. Studies in brain slices (Zonta et al., 2003; Mulligan and MacVicar, 2004; Gordon et al., 2008) as well as *in vivo* (Takano et al., 2006; Petzold et al., 2008; Mishra et al., 2011; Srienc et al., 2012) have led to the disentanglement of the complex mechanisms that rule blood microcirculation, in which astrocyte calcium signal play a prominent role mediating neuron-glia-vascular coupling (Attwell et al., 2010).

**FIGURE 4:**
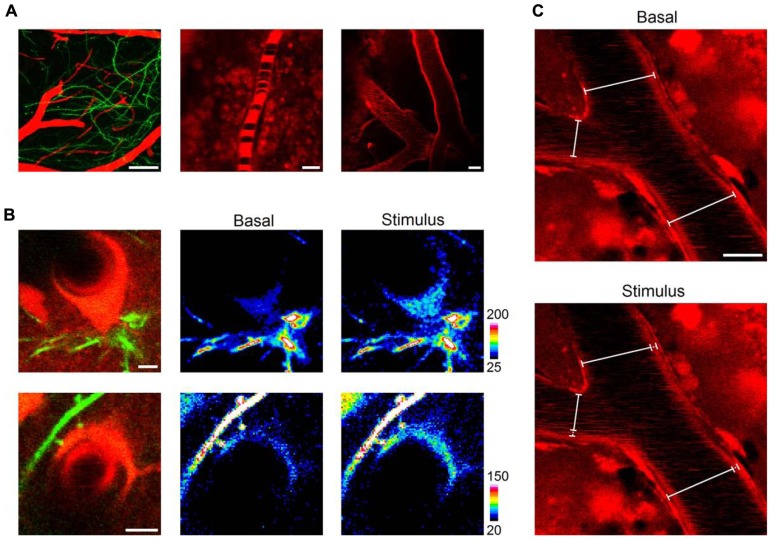
***In vivo* imaging of blood vessel dynamics**. Shortly after (~20 min) a single intraperitoneal injection, SR101 distinctly evidenced cortical blood vessels intermingled with neuronal dendrites (**A**, left panel, 50 μm z-stack projection). Erythrocytes were clearly observed circulating as dark bodies inside blood vessels of different diameters (5–50 μm; **A**, central and right panels). Astrocytic somata and endfeet were observed enwrapping blood vessels (**B**, upper and lower panel, respectively) and showed calcium elevations upon sensory stimulation (**B**, central and right panels). The distinct staining of blood vessels allowed us to monitor changes in diameter throughout the stimulation protocol **(C)**. Scale bars: 50, 5, and 20 μm for left, central, and right panels in **(A)**; 5 μm in **(B)**; 10 μm in **(C)**.

After observing that we could reliably monitor astrocytes and astrocytic-related structures along with subcellular calcium transients, we aimed to image blood vessel dynamics *in vivo* with LSCM after intraperitoneal injection of SR101. Indeed, when monitoring the barrel cortex, we were able to observe pial blood arteries penetrating into the brain accompanied by a net of dendrites arising from layer V neurons (Feng et al., 2000; **Figure 4A**). We observed blood vessels of several diameters (range 5–50 μm) and negatively contrasted erythrocytes, which were not stained with SR101 but observed as dark cell bodies over the background (**Figure 4A**). This was especially evident in the first minutes to 2–3 h after the intraperitoneal injection of SR101, when most of the dye was cleared from the blood. Also, as mentioned above, we observed characteristic astrocytic structures adjacent to and enwrapping blood vessels with their soma or endfeet which showed calcium increases upon sensory stimulation (**Figure 4B**). We then monitored blood vessel diameter in response to sensory stimulation. After imaging basal conditions (30–45 s), we observed that delivery of whisker-tail stimulus (see above) for 30 s induced a change in inner and outer diameter a few seconds (~4 s) after the onset of the stimulus (**Figure 4C**).

These results, which are in agreement with previous reports using LSCM (Villringer et al., 1989) or 2PLSM (Kleinfeld et al., 1998; Takano et al., 2006), indicate that the combination of SR101 injection and LSCM is suitable to probe functional changes in blood vessel diameters and blood flow dynamics. Furthermore, erythrocytes were observed circulating inside blood vessels and although we did not study erythrocyte velocity, this could be easily achieved employing the line scan mode of LSCM (Dirnagl et al., 1991; Kleinfeld et al., 1998).

## DISCUSSION

In the present article we show the suitability of LSCM to monitor and assess important characteristics of astrocyte structure and function *in vivo*, i.e., astrocyte morphology, subcellular structural interactions between astrocytic processes and dendritic spines, calcium dynamics in astrocytic soma and processes, and changes in blood vessel diameter and blood flow dynamics. Additionally, we provide a guide that details the experimental steps used to attain these *in vivo* recordings.

While our study focused on somatosensory cortex, where cells could be directly imaged from the brain surface due to their relatively superficial location, deep imaging of the cortex without removing superficial layers can only be achieved using 2PLSM. Nevertheless, our procedure may be extended to study different brain structures including the hippocampus, where special surgical procedures have been employed to remove the cortical overlying area and obtain *in vivo* recordings from neurons and astrocytes (Mizrahi et al., 2004; Navarrete et al., 2012).

The classical view of astrocytic function attributed to them a simple supportive role to maintain the homeostatic conditions for the proper function of neurons. However, a novel view of astrocyte function in brain physiology has emerged, i.e., the tripartite synapse concept, where astrocytes actively interact with neurons and are integral elements of synaptic physiology. This concept implies an active role of astrocytes in brain function, which would hence result from the concerted activity of astrocytes and neurons.

According to this new concept, astrocyte calcium signal is a key element because it is the second messenger that serves as substrate of astrocyte excitability underlying its responsiveness to neurotransmitter release during synaptic activity. It stimulates the release of gliotransmitters that regulate synaptic transmision and plasticity (for reviews see Araque et al., 1999; Volterra and Meldolesi, 2005; Perea et al., 2009). While most of the reports supporting this new concept originally derived from studies performed in cell cultures (Araque and Perea, 2004) and slices (Perea et al., 2009), more recent work are also based on *in vivo* studies. Indeed, astrocyte calcium elevations evoked by electrical or sensory stimulation have been reported in the hippocampus (Navarrete et al., 2012), somatosensory barrel cortex (Wang et al., 2006; Takata et al., 2011), and visual cortex (Schummers et al., 2008; Chen et al., 2012) *in vivo*. Present results show that astrocytes located in the layer I of the primary somatosensory cortex also respond to sensory stimuli, further supporting the astrocyte responsiveness to synaptic activity evoked by sensory inputs *in vivo*.

Interestingly, while previous *in vivo* astrocytic calcium transients were recorded at the soma (Hirase et al., 2004; Wang et al., 2006; Schummers et al., 2008; Takata et al., 2011; Chen et al., 2012; Navarrete et al., 2012), strong evidence obtained in slices indicate that the physiologically relevant calcium signal may occur at discrete regions -microdomains- of the astrocytic processes (Grosche et al., 1999; Perea and Araque, 2005; Di Castro et al., 2011; Panatier et al., 2011). Our present results indicate that astrocyte calcium elevations evoked by sensory stimuli *in vivo* also take place at regions of the fine processes closely associated with dendritic spines, i.e., where most synapses are established with excitatory terminals.

A recent report has questioned the validity of the tripartite synapse concept in adult animals based on the absence of mGluR5 expression in adult astrocytes and mGluR5-mediated astrocyte calcium elevations (Sun et al., 2013). However, the reported absence of mGluR5-mediated calcium responses in adults discards this particular mechanism of astrocytic activation, but the calcium responsiveness of astrocytes through different signaling mechanisms and to different synaptically released neurotransmitters [e.g., glutamate, acetylcholine (ACh), endocannabinoids, adenosine triphosphate (ATP), norepinephrine, etc.] is strongly supported by numerous evidence obtained in slices and *in vivo* (Wang et al., 2006; Navarrete and Araque, 2008, 2010; Schummers et al., 2008; Di Castro et al., 2011; Panatier et al., 2011; Takata et al., 2011; Chen et al., 2012; Min and Nevian, 2012; Navarrete et al., 2012; for a review see Perea et al., 2009). On the other hand, the tripartite synapse concept based on the ability of astrocytes to release gliotransmitters that regulate synaptic transmission and plasticity is also supported by abundant evidence obtained by numerous laboratories (Perea and Araque, 2007; Fellin et al., 2009; Henneberger et al., 2010; Navarrete and Araque, 2010; Di Castro et al., 2011; Panatier et al., 2011; Min and Nevian, 2012). Regardless the underlying molecular mechanisms, our present results obtained in adult animals *in vivo* add further support of the idea that astrocytes respond with calcium elevations to sensory stimuli.

Simultaneous imaging of astrocyte calcium and blood vessels in slices as well as *in vivo* has provided relevant information to decipher the complex mechanisms controlling cerebral blood flow microcirculation (Zonta et al., 2003; Mulligan and MacVicar, 2004; Takano et al., 2006; Gordon et al., 2008; Petzold et al., 2008; Srienc et al., 2012; for a review see Attwell et al., 2010). Notably, Eric Newman’s lab has developed an intact *in vivo* preparation in which the retina of anesthetized, paralyzed rats can be directly imaged with LSCM and laser speckle flowmetry to monitor retinal glial cell responses and retinal blood flow (Mishra et al., 2011; Srienc et al., 2012). Using this intact preparation they were able to demonstrate that light stimulation evoked glial calcium waves that led to the dilation of neighboring retinal arterioles, indicating that glial cells respond to sensory stimuli and subsequently regulate blood flow *in vivo*. In agreement with these reports, our results show that sensory stimulation leads to calcium elevations in astrocytic somata and endfeet enwrapping blood vessels along with changes in blood vessel diameter in the somatosensory cortex*.* Furthermore, they confirm the suitability of our method to study blood flow microcirculation *in vivo*, prompting further studies regarding blood flow control in healthy as well as pathological conditions in the live animal.

## Conflict of Interest Statement

The authors declare that the research was conducted in the absence of any commercial or financial relationships that could be construed as a potential conflict of interest.
